# Smart Packaging Based on Polylactic Acid: The Effects of Antibacterial and Antioxidant Agents from Natural Extracts on Physical–Mechanical Properties, Colony Reduction, Perishable Food Shelf Life, and Future Prospective

**DOI:** 10.3390/polym15204103

**Published:** 2023-10-16

**Authors:** Halimatuddahliana Nasution, Hamidah Harahap, Elisa Julianti, Aida Safitri, Mariatti Jaafar

**Affiliations:** 1 Department of Chemical Engineering, Faculty of Engineering, Universitas Sumatera Utara, Padang Bulan, Kec. Medan Baru, Medan 20155, Sumatera Utara, Indonesia; hamidah.harahap@usu.ac.id (H.H.); aidasafitri853@gmail.com (A.S.); 2 Department of Food and Science Technology, Faculty of Agriculture, Universitas Sumatera Utara, Padang Bulan, Kec. Medan Baru, Medan 20155, Sumatera Utara, Indonesia; elisa1@usu.ac.id; 3 School of Materials & Mineral Resources Engineering, Universiti Sains Malaysia, Nibong Tebal 14300, Pulau Pinang, Malaysia; mariatti@usm.my

**Keywords:** colony reduction, food shelf life, mechanical properties, natural additive, PLA, smart packaging

## Abstract

Changes in consumer lifestyles have raised awareness of a variety of food options and packaging technologies. Active and smart packaging is an innovative technology that serves to enhance the safety and quality of food products like fruit, vegetables, fish, and meat. Smart packaging, as a subset of this technology, entails the integration of additives into packaging materials, thereby facilitating the preservation or extension of product quality and shelf life. This technological approach stimulates a heightened demand for safer food products with a prolonged shelf life. Active packaging predominantly relies on the utilization of natural active substances. Therefore, the combination of active substances has a significant impact on the characteristics of active packaging, particularly on polymeric blends like polylactic acid (PLA) as a matrix. Therefore, this review will summarize how the addition of natural active agents influences the performance of smart packaging through systematic analysis, providing new insights into the types of active agents on physical–mechanical properties, colony reduction, and its application in foods. Through their integration, the market for active and smart packaging systems is expected to have a bright future.

## 1. Introduction

The rise in plastic food packaging waste due to the large number of industries involved in the production of fast food has led several countries to encourage their packaging industries to improve the efficiency of the food supply chain in order to reduce food spoilage and waste. To address this situation, the incorporation of active agents such as antimicrobial and antioxidant compounds into packaging materials has emerged as a viable solution for extending food shelf life, reducing food losses, and increasing food industry profitability [1,2,3]. Active packaging, also known as smart packaging, is designed to detect and alert producers to spoilage or other potential problems in packaged food [4,5,6]. These systems, which are classified as direct (humidity, time–temperature, freshness, damage, and biosensor) and indirect (traceability and tracking), serve as quality indicators to ensure food safety [7,8,9].

In order to maintain the product’s nutrients, protection, and quality throughout the distribution chain and to ensure that it reaches consumers for final use and consumption, it is crucial to extend the shelf life of food products through the control of microbial and chemical processes both inside the product and on the product’s surface [10]. Food can be stored and kept fresh for a long time in packaging with high barrier properties, which make it impermeable to gases and moisture [11,12]. High barrier properties also prevent chemical oxidation and lower microbial spoilage, which is primarily caused by the presence of aerobic microorganisms. The information that markers provide on microorganism activity varies due to chemical differences, reactions, or microbiological developments that occur as a result of time and processing. When metabolites produced by microbial growth interact with chemical compounds, they produce both a visual signal and information about degradation [13,14].

**Figure 1:**
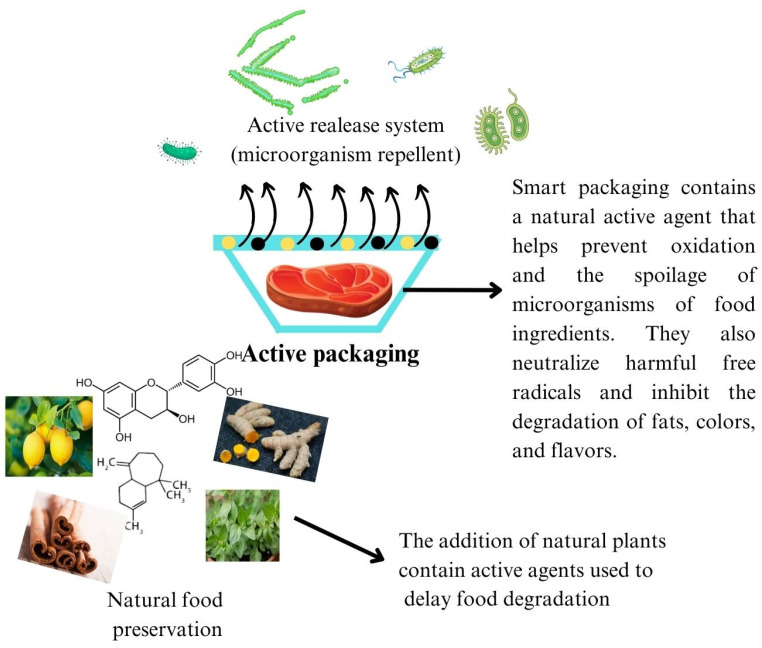
The role of various natural active agents in maintaining food shelf life in smart packaging systems.

Utilizing plant extracts like curcumin, garlic, tea, and propolis in active packaging systems presents a multifaceted approach for the extension of the shelf life of food ingredients. As shown in Figure 1, their antioxidant properties prevent oxidation, their antibacterial properties reduce microbial growth, and their active release mechanisms ensure a continuous protective effect. The anti-bacterial and antioxidant food packaging system is currently being developed to examine interactions among food components, packaging, and the environment in order to improve product quality, safety, and shelf life [15,16,17]. However, the deployment of antimicrobial and antioxidant agents as packaging materials must rigorously adhere to established guidelines, particularly those concerning toxicological repercussions. Antibacterial agents of various types, including organic synthetic antibacterial agents, inorganic antibacterial agents, and natural antibacterial agents, are now used in food preservation [18,19,20]. The three types of natural antibacterial agents are animal-derived antibacterial agents (such as protamine, propolis, and chitosan), microbial-derived antibacterial agents (such as lysozyme, nisin, and natamycin), and plant-derived antibacterial and antioxidant agents (such as plant essential oils, tea polyphenols, and Chinese herbal medicines) [21,22]. 

**Figure 2:**
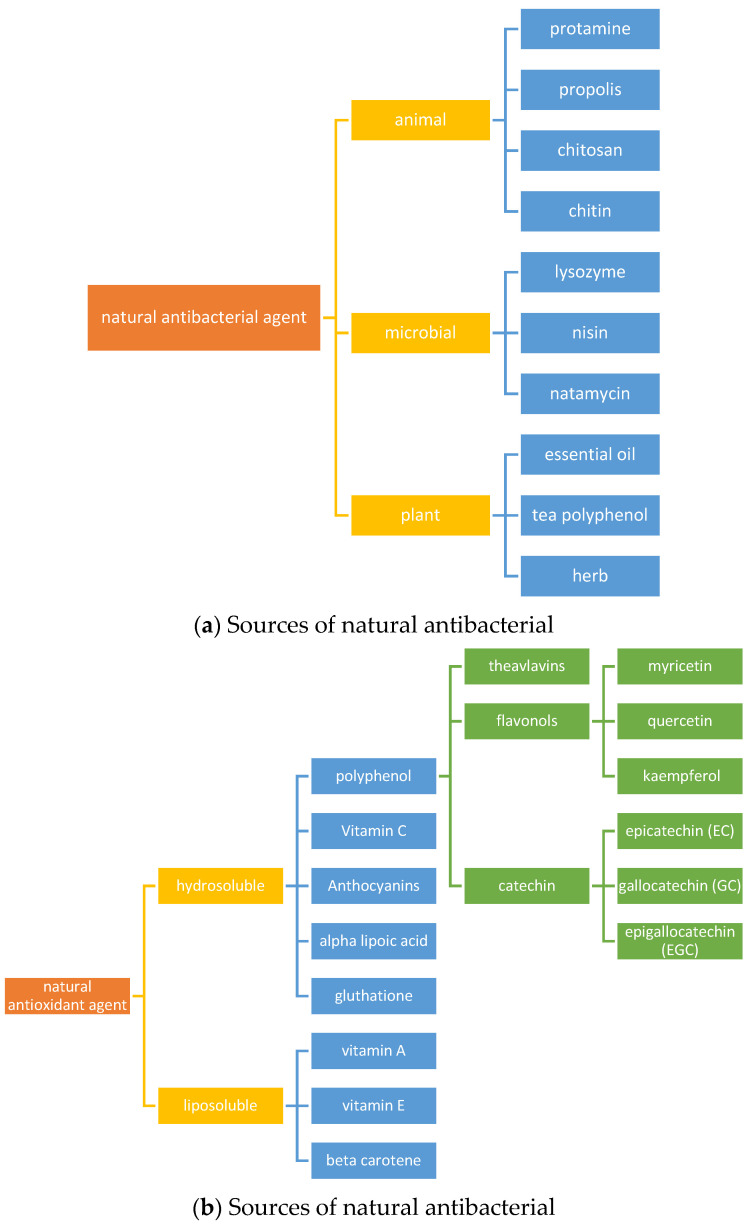
Common antibacterial (**a**), and (**b**) antioxidant agents in smart packaging.

Natural antimicrobial and antioxidant agents are extracted and purified from raw natural sources, as depicted in Figure 2. This is due to their chemical constituents, encompassing compounds like anthocyanins, catechins, vitamin A, and beta-carotene. Additionally, plant-derived materials mostly possess both antibacterial and antioxidant characteristics [23,24,25]. These constituents are frequently employed in the formulation of active packaging materials. As packaging systems with active features undergo diverse storage and processing circumstances, the degradation of food items within can lead to the creation of metabolites like volatile amines and organic acids; thus, plant extracts are deemed safer compared to synthetically produced preservatives due to their origin as secondary metabolites within plants, as indicated in Table 1. Moreover, the robust antibacterial activity exhibited by plant extracts has been demonstrated, effectively inhibiting a range of foodborne pathogens such as *E. coli*, *Salmonella typhi*, *Staphylococcus aureus*, and *Bacillus cereus*. This serves to affirm their enhanced versatility across a spectrum of applications [26,27,28]. 

Nowadays, there is a high demand in the consumer market for healthy, organic, and wholesome products with a “clean” label. As a result, research into smart packaging to improve the quality and safety of food ingredients is critical. Therefore, food packaging has become part of modern civilization and is developed using biopolymers materials. A biopolymer is an organic polymer containing monomeric units of an organic substance that are covalently linked together. It possesses biodegradability, which means it can be naturally broken down into the soil by microorganisms, and it emits organic byproducts such as CO_2_ and H_2_O that are beneficial to the environment. Therefore, disparities in the biodegradability and composting potential of biodegradable plastics play a vital role in promoting environmentally friendly disposal practices. One crucial difference lies in the fact that biodegradable plastics can break down under specific circumstances and within different timeframes [29].

In the context of active packaging, a range of polymers have been utilized as matrixes for developing innovative solutions. Natural biopolymers, sourced from renewable materials, include starch, cellulose, chitosan, and proteins, which are known for their inherent biodegradability and compatibility with living systems. Conversely, synthetic polymers such as polyethylene, polypropylene, and polyethylene terephthalate offer adaptable mechanical properties and high barrier capabilities [30,31,32]. Furthermore, chemically engineered synthetic biodegradable polymers like polylactic acid (PLA), polyhydroxyalkanoates (PHA), polybutylene adipate terephthalate (PBAT), polyglycolic acid (PGA), and polyvinyl alcohol (PVA) present customized degradation patterns, harmonizing ecological considerations with packaging effectiveness. This diverse array of polymers, encompassing both natural and synthetic origins, forms the cornerstone for active packaging systems endowed with a variety of functions aimed at augmenting product shelf life, ensuring safety, and addressing environmental concerns [33]. Whereas biopolymers are completely obtained from renewable resources, these are manufactured from non-renewable resources (fossil-sourced chemicals). Despite extensive efforts to enhance their properties using various techniques, biopolymer-derived materials frequently lack the performance characteristics of traditional plastics in terms of strength, flexibility, and barrier qualities [34,35].

PLA is one of the most promising biopolymers for a variety of food applications, and it can be converted into smart packaging through commercial manufacturing processes [36]. PLA is frequently suggested as a raw material for packaging and beverages because it offers better mechanical strength and durability and has a good appearance compared with other polymers such as polyurethane, polystyrene, and polypropylene [37,38]. PLA has several desirable properties, including high transparency, clarity, and insolubility with air, ethanol, methanol, and aliphatic carbon [39]. The main disadvantages of PLA, especially for flexible food packaging applications, are its brittleness and heat distortion temperature, as well as its low water vapor barrier properties [40,41]. 

**Figure 3:**
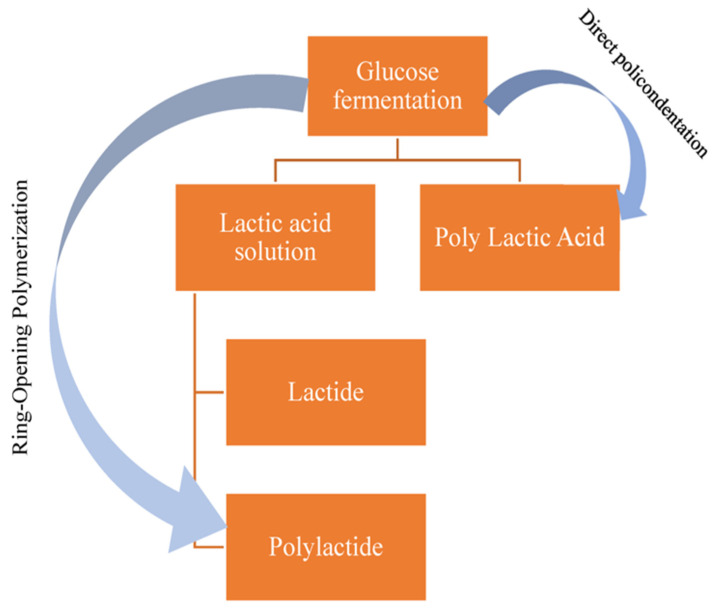
Methods to produce PLA.

PLA can be made using two common methods as shown in Figure 3: Direct Polycondensation (DP) and Ring-Opening Polymerization (ROP) [42].

Many researchers have previously investigated the development of active packaging based on PLA containing antioxidant and antimicrobial active agents. A study by [43] evaluated PLA blends containing 3% and 6% thymol to produce smart packaging films for antifungal activity against *Aspergillus* spp. and *Penicillium* spp. The addition of thymol significantly increased the thermal and barrier properties of the film, allowing it to extend the shelf life of bread packaging by up to 9 days compared to commercial polypropylene plastic [43]. Further study by [44] also produced a different film that was UV blocking and reduced microbial activity. Modification was made with the addition of curcumin so that the packaging film showed significant antibacterial activity against *E. coli*. The packaging films also had strong UV inhibition capabilities and physical properties. Another study by [45] also produced active films using additives made from *Syzygium cumini* peel extract. The 2,2-azinobis-3-Ethylbenzothiazoline-6-Sulfonic Acid (ABTS) and 2,2-diphenyl-1-picrylhydrazyl (DPPH) methods were used to verify the samples’ antioxidant activity. Due to the high concentration of phenolic hydroxyl groups in the film’s structure and the addition of more than 30% extract, the antioxidant properties of the film were increased, enabling it to scavenge free radicals by donating phenolic hydrogen atoms, thus improving food shelf life. 

**Figure 4:**
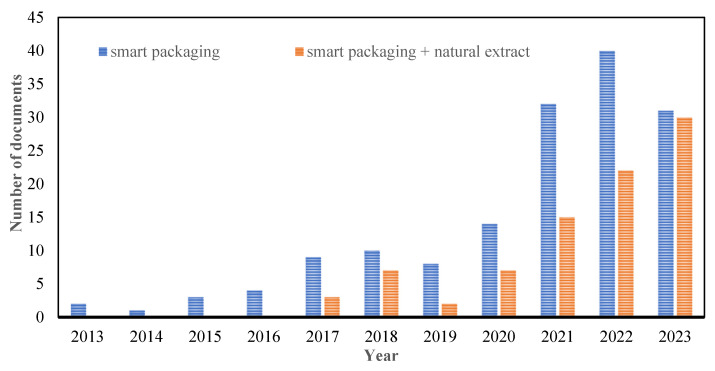
Scopus database from 2013–2023 with the keywords “Smart packaging” and “Smart packaging + natural extract”.

Drawing from the Scopus database based on Figure 4, it is evident that over the past decade, the field of smart packaging has evolved into a nascent discipline within the development of packaging. This surge in interest within the smart packaging sector has risen by an impressive 140%. This advancement notably revolves around the exploration of natural extracts and is termed “smart packaging + natural extract”. However, until the present, other studies have solely focused on the single properties of packaging materials or bioactive agents, but this review uniquely combines these elements to reveal a synergistic relationship. By investigating the intricate interplay between PLA and antibacterial/antioxidant agents derived from natural extracts, this paper delves into uncharted territory, exploring how the combined influence of these components not only influences the physical–mechanical characteristics of the packaging material but also its efficacy in reducing microbial colonies and preserving perishable foods.

The novelty of this review paper lies in its ability to present a holistic perspective, showcasing how the introduction of antibacterial and antioxidant agents from natural sources can bring multifaceted improvements to PLA-based smart packaging. This study underscores the profound impact of these agents on the packaging material’s strength, flexibility, and barrier properties, while concurrently unveiling their potential to inhibit microbial growth and oxidative deterioration. Moreover, this review paper transcends traditional boundaries by pioneering a comprehensive examination of PLA-based smart packaging as an integrated ecosystem. As industries worldwide seek sustainable and effective packaging strategies, the findings and insights presented in this paper herald a significant advancement, setting a precedent for future research at the crossroads of materials in science, biochemistry, and food technology.

## 2. The Effects of Antibacterial and Antioxidant Agents from Natural Extracts on Physical–Mechanical Properties

### 2.1. Tensile Properties

The term “rough handling” emphasizes the importance of tensile strength in the plastic packaging industry, which determines its ability to withstand and protect itself from external pressure. Therefore, the tensile test is one of the most important parameters for evaluating the mechanical performance of polymer blends, particularly in the production of smart packaging. Tensile strength is the maximum force that a material can withstand, and elongation at break is where the material’s extensibility is measured. Among all biopolymers, PLA—an aliphatic polyester derived from renewable resources, specifically starch fermentation—caught the interest of researchers as a potential packaging material. 

In spite of possessing commendable mechanical, thermal, and biodegradable characteristics, their practical applications encounter limitations stemming from inadequate flexibility, limited impact resilience, suboptimal barrier properties, and a constrained processing range. Attempts to enhance these characteristics have been undertaken through diverse methodologies, including blends with alternative biopolymers, chemical adaptations, and the incorporation of responsive additives [46,47]. As depicted in Table 2, variations in the tensile properties of intelligent packaging based on PLA hinge on the specific active agent employed. Typically, the tensile strength of PLA blends spans approximately 40 MPa to 70 MPa. Evidently showcased in Table 2 is the noteworthy decline in all resultant tensile strength values. The active agent, which is from the essential oil group, decreases the tensile strength value due to a heterogeneous internal structure with lower cohesiveness [48,49]. The tensile strength of the resulting film also decreases due to the plasticizing effect of essential oils. Essential oils are highly hydrophobic, so they affect the hydrophilic/hydrophobic balance of the film [50,51]. Based on the previous works, it can be concluded that the tensile properties of PLA smart packaging are influenced by the amount of active agent, type of active agent, and specific formulation of the PLA blend.

Essential oils can also reduce the oxygen permeability of the film by forming a more porous microstructure. The mechanical properties of the film modify due to the development of structural discontinuities, which results in flexibility and lower resistance to cracking. Elongation at break shows a different pattern. The incorporation of PLA with essential oils into the film results in a slight increase in the data’s average elongation at break value.

The increased elongation at break observed in the films is also a result of the essential oil loading’s plasticizing effect, which reduces stiffness and increases film flexibility by allowing more chain mobility. However, essential oil concentrations greater than 10% by weight cause an antiplasticization phenomenon in which the interaction between the plasticizer and polymer molecules is stronger, inhibiting macromolecule mobility and leading to a very brittle film [52,53,54]. The addition of anthocyanin-rich plant extracts such as pomegranate also reduced the film’s tensile strength, but only by 15–20%, because it was able to maintain denser film through interfacial adhesion [55,56,57]. Thus, when producing smart packaging, the chosen combination of polymeric materials and active agents must have similar properties in order to achieve better interfacial adhesion. Thus, using a hydrophilic polymer matrix and hydrophilic agents—or hydrophobic and hydrophobic—results in a strong bond between the materials [58]. A matrix and active agents with similar properties also imply better dimensional stability and maintain their mechanical properties.

When conducting research, it might be challenging to figure out the appropriate quantity of active agent to add in order to achieve optimal interaction between the additive and the matrix while avoiding phase separation and filler particle agglomeration. Table 2 also shows that the addition of an active agent between 0.5% wt and 20% wt has plasticizing properties because it contains a lot of aromatic ring structures that inhibit the polymer network from being arranged closely, providing more flexibility and higher elongation at break value. By weakening the chain’s structure, the plasticizing effect also reduces cohesiveness and increases deformability and flexibility, partially replacing the stronger polymer–polymer interactions. Unless nanofiber is added, which can lengthen polymer chains, as research by [59] found, nanofibers that have outside forces, such as rigidity and durability of the film, provide in situ polymerization and come to form covalent bonds while monomers or polymer chains interact with the filler materials.

### 2.2. Water Vapour Transmission Rate (WVTR)

The WVTR value is a standard measure of how easily moisture can penetrate the film, the packaging’s ability to withstand different humidity levels at different temperatures, and the ability to keep the quality of the food ingredients inside until it reaches the consumer. For food products, moisture migration can lead to undesirable texture changes or a loss of flavor. Controlling moisture through proper packaging helps preserve the product’s sensory qualities. Moisture also causes packaging materials to warp, labels to detach, and colors to fade, affecting the overall appearance and appeal of the product; therefore, a consistent WVTR value ensures that products maintain a consistent weight, volume, and overall quality, helping manufacturers deliver products that meet consumer expectations. Typically for solid polymers, the transmission of water vapor follows a simple mechanism whereby water vapor penetrates the film by adsorbing on the surface and dissolving rapidly, thereby establishing an equilibrium that spreads through the film and causes desorption on the surface.

The use of PLA as a potential material for food packaging is greatly limited by the higher water vapor transmission rate (WVTR) of the films. Since PLA-based films indicate high WVTR, according to previous studies, strengthening strategies using natural extracts and essential oils have been known to improve the barrier properties of PLA-based films. However, based on Table 3, the additive incorporation must be considered because it changes the balance of the film’s hydrophilicity and hydrophobicity [60,61]. The main factors that influence WVTR are differences in the physical properties of the matrix and additive, operating conditions, the diffusion coefficient, the solubility of water molecules, and the three-dimensional structure formed by hydrogen bonding [62]. The types of molecules and the compatibility of additives with the matrix are important factors affecting dispersion and physical and/or chemical interactions with the polymer matrix, along with chemical structure and polarity. The WVTR value in the film, however, is also influenced by other variables, including the crystallinity of the polymer, as well as the absorption of molecules inside in the matrix [63,64,65].

## 3. The Effects of Antibacterial and Antioxidant Agents from Natural Extracts on the Microstructure of Smart Packaging

The microstructure of smart packaging materials can be engineered to create effective barriers against external factors such as moisture, oxygen, light, and contaminants [78]. By carefully tailoring the microstructure, packaging materials can prevent the ingress of these detrimental elements, thereby safeguarding the sensory characteristics, nutritional value, and overall quality of the packaged food. This preservation is especially important for perishable and sensitive products. Smart packaging’s microstructure can be designed to slow down the deterioration processes that occur in food over time [79]. For instance, incorporating oxygen-absorbing or moisture-absorbing materials at the microstructural level can reduce the rate of oxidative reactions and microbial growth, effectively extending the shelf life of the product. This is not only economically beneficial but also contributes to reducing food waste.

**Figure 5:**
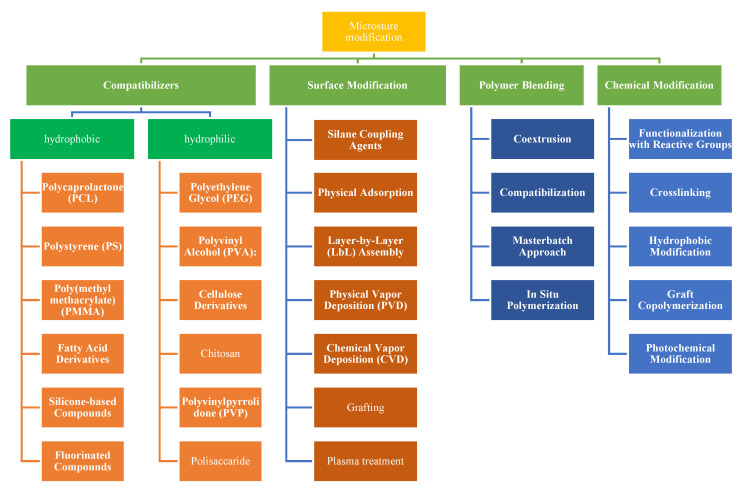
Microstructure modification of smart packaging based on PLA.

Integrating active agents within the microstructure of smart packaging allows for controlled and targeted release. This is particularly advantageous when active compounds such as antimicrobial agents or antioxidants are incorporated. The microstructure can facilitate the gradual release of these compounds, providing continuous protection against spoilage microorganisms and oxidative reactions, thereby maintaining food safety and quality [80]. A common attempt has been used by previous researchers to enhance compatibility and facilitate interactions between polymeric blends in the production of smart packaging systems (Figure 5).

Microstructure modification also encompasses changing the configuration and characteristics of materials at the microscopic scale with the aim of attaining targeted enhancements in the functionality of food packaging. This process has the potential to augment properties such as barrier capabilities, adhesion, compatibility, and the holistic performance of the packaging system. The roles of compatibilizers, surface modification, polymer blending, and chemical modification are as follows:

- Compatibilizers are additives used to improve the compatibility between two or more polymers with differing properties. In food packaging, where different polymers may need to work together, compatibilizers help create a cohesive structure and improve properties like adhesion, mechanical strength, and barrier performance. Compatibilizers achieve this by promoting interfacial interactions between polymers that would otherwise induce phase separation or have weak interactions.
- Surface modification encompasses the adjustment of material surface characteristics to amplify adhesion, wettability, and harmonization with additional substances. Surface modification assumes paramount significance in optimizing the interplay between packaging materials and the contents. Methodologies such as plasma treatment, layer-by-layer (LbL) assembly, and chemical grafting engender the introduction of functional groups onto the surface, fostering an augmented propensity for adhesion or coating. This in turn elevates the packaging material’s barrier properties, print quality, and holistic performance.
- Polymeric blending techniques offer effective ways to improve adhesion and compatibility between hydrophilic or hydrophobic polymer materials in smart packaging systems.
- Chemical modification involves changing the chemical structure of the polymer to achieve desired properties. Functional groups can be introduced to improve compatibility, adhesion, or specific interactions. In food packaging, chemical modification can adapt the properties of the packaging material to meet specific requirements.

Surface modification has been extensively explored and applied in active packaging research, as is evident from existing publications. Surface modification techniques offer a wide range of applications in active packaging (enhancing barrier properties, incorporating functional groups for controlled release or antimicrobial effects, minimizing disruption to the overall structure while significantly improving adhesion, and compatibility). Surface modification also complements other techniques, such as incorporating antimicrobial agents, antioxidants, or moisture absorbers. Therefore, component selection and blending methods should be considered in order to achieve desired compatibility while maintaining the essential properties of the packaging material. In-depth characterization and testing are crucial to ensure the successful integration of blended polymers in functional packaging solutions.

## 4. The Effects of Antibacterial and Antioxidant Agents from Natural Extracts on Colony Reduction 

The use of antibacterial agents is paramount for thwarting the formation of biofilms, which are intricate microbial communities that adhere to surfaces and are enveloped within a protective matrix. This multifaceted process initiates with the attachment of bacteria to a surface, setting the stage for biofilm development. Active components present in substances such as essential oils and plant extracts, for instance, possess the capability to modify the structural composition of bacterial cell membranes. A study by [81] indicated that the transformative action renders bacterial attachment to surfaces more challenging. Moreover, the biofilm matrix, comprising extracellular polymeric substances (EPS), furnishes a safeguarding shield for bacteria within the biofilm community. This defensive layer is susceptible to degradation or disruption by antibacterial agents, thereby compromising the biofilm’s structural integrity and rendering it more vulnerable to removal.

Staphylococcus aureus, Listeria monocytogenes, Escherichia coli O157:H7, and Salmonella Typhimurium are sources of biofilm-forming bacteria behind global instances of foodborne illnesses [82]. These virulent microorganisms can contaminate a spectrum of foods, spanning from ready-to-eat vegetables to processed meat products. There is mounting concern in public health circles about these microorganisms due to their stature as enteric pathogens. Associated with worldwide afflictions like diarrheal disease, peritonitis, colitis, bacteremia, infant mortality, and urinary tract infections, these pathogens inflict substantial economic burdens due to treatment costs. In the context of biofilm growth, where bacteria adhere to surfaces, an avenue to curtail the pathogenic influence of Gram-positive bacteria involves impeding their adherence to both living and non-living surfaces [83]. 

**Figure 6:**
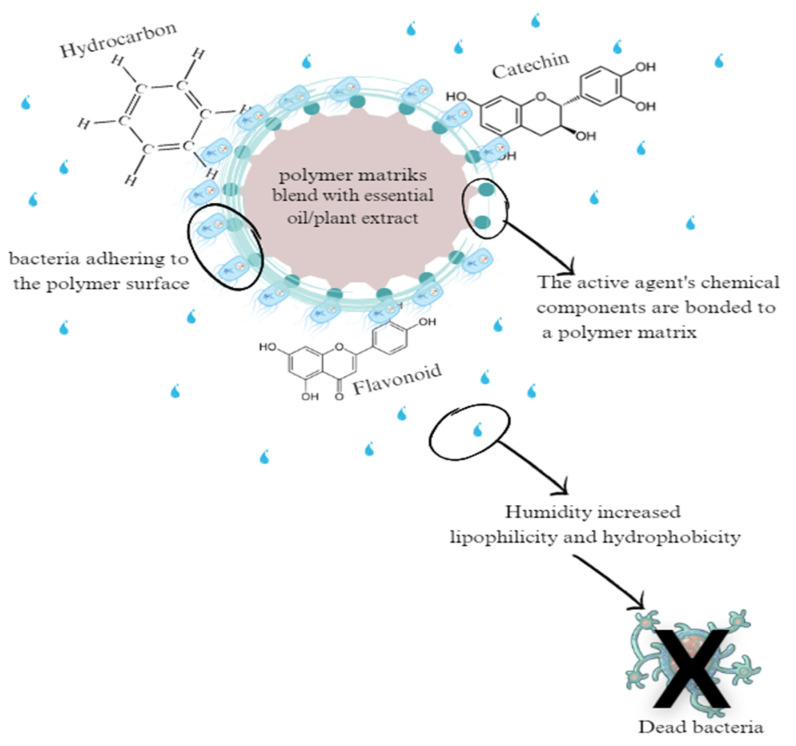
Dead cell mechanism.

Usually in food spoilage, an active agent is released into the Staphylococcus aureus, Listeria monocytogenes, Escherichia coli O157:H7, or Salmonella typhimurium membrane structure due to the presence of moisture in the air, which increases lipophilicity and hydrophobicity, which then causes membrane expansion, increased membrane fluidity and permeability, disruption of membrane-embedded proteins, inhibition of respiration, and changes in bacterial ion transport processes. The active agent destroys the bacterial cell membrane and binds directly to DNA gyrase, as depicted in Figure 6. DNA gyrase is an essential part of bacteria that plays an important role in the replication of DNA and chromosomal segregation. One of the most extensively investigated mechanisms for killing bacteria is the inhibition of DNA gyrase. The antibacterial activity of a group of chemical substances that consist of flavonoids, hydrocarbons, and catechins interact with the outermost layer of the protein of bacteria, thereby preventing its growth.

Secondary metabolites such as alkaloids, flavonoids, steroids, saponins, terpenoids, and tannins are likely to be responsible for this antibacterial activity in plant extracts and essential oils [84]. It is important to remember that the compatibility of hydrophilic compounds and hydrophobic matrices can influence the effectiveness of antimicrobial properties. As shown in Table 4, the incorporation of plant extracts prevails over essential oils in terms of bacterial colony reduction. Additionally, it was discovered that due to variations in the structure of the bacterial cell wall and outer membrane, Gram-positive bacteria were more susceptible to PLA films containing active essential agents than Gram-negative bacteria [85]. 

## 5. The Effects of Antibacterial and Antioxidant Agents from Natural Extracts on Natural Perishable Food Shelf Life

Foods are perishable because they have a short shelf life and are extremely sensitive to factors like humidity, temperature, and other factors. The refrigerator has prolonged the shelf life of perishable food up until this point, but food deterioration is unavoidable. Nowadays, improving packaging systems has become essential for preserving the quality of food ingredients. Bacterial biofilm formation is regarded as a newly emerging microbial lifestyle that thrives on all types of surfaces and is present in both natural and artificial environments. 

**Figure 7:**
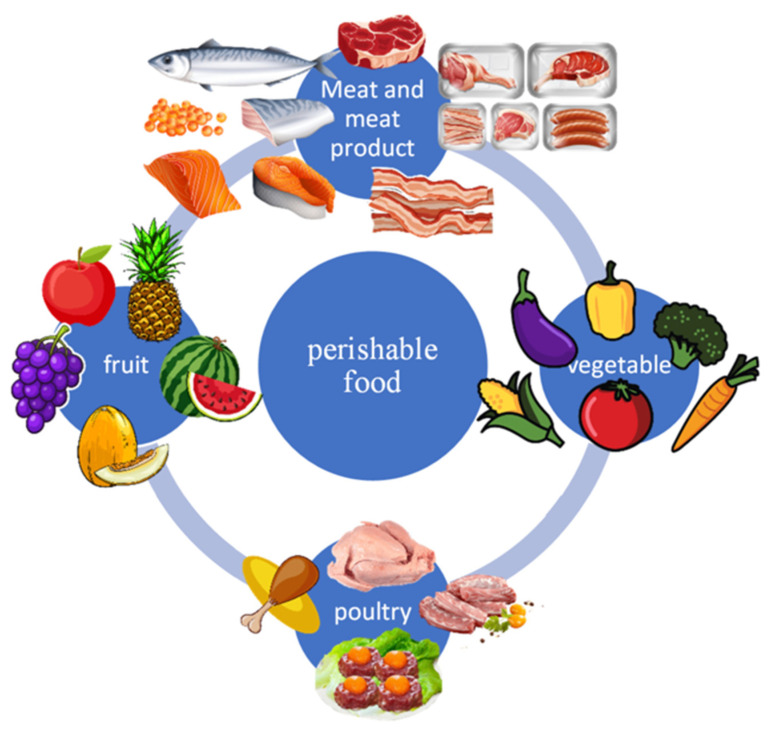
Types of food that are susceptible to microorganisms.

As shown in Figure 7, meat, poultry, egg products, salads, tuna, chicken, potatoes, and macaroni are the main foods that are commonly infected with bacteria [96]. Foods rich in protein tend to be decomposed by bacteria. A Gram-positive bacterium can attach to glass, metal, and plastic as an abiotic surface and host tissue as a biotic surface [97]. The attachment of those bacteria to surfaces depends on components of the bacterial microbial surface that recognize adhesive matrix molecules for proteins. To prevent attachment to the surface through the matrix, the surface must be coated with anti-adhesion agents such as arylrhodamines, calcium chelators, essential oils, plant extracts, silver nanoparticles, and chitosan [98].

Even when optimal conditions are provided during distribution, agricultural products have a short shelf life from the time of harvest onward, due to quality degradation between harvest and consumption. If the product is not handled properly, this loss in quality could be significant. Quality is a key marketing component that is becoming increasingly crucial for both producers and consumers. Therefore, quality management is crucial in the distribution of agricultural products. Definitions of quality have been developed in various research fields as a result of this growing significance. It is inevitable for foodborne pathogens to form biofilms, which can contaminate food. There have been numerous studies, as shown in Table 5, that investigate the use of natural ingredients as natural preservatives that are safe for use in packaging systems.

As can be seen in Table 5, the addition of active agents can increase the shelf life of food ingredients (33% TBARS reduction in aldehyde) in food samples packaged with active films for 12 days to 30 days, since lipid oxidation is one of the most important processes causing the deterioration of meat and meat products. Aldehydes, ketones, and alcohols are just a few of the volatile and nonvolatile compounds that are produced when the lipids in meat oxidize [106]. These compounds provide meat its rancidity, taste, odor, and color loss. One of the most important indicators for assessing the freshness of meat and meat products is TVB-N content. TVB-N is primarily made up of ammonia (NH_3_), dimethylamine, trimethylamine, putrescine, and cadaverine, which are created when putrefactive microorganisms break down protein and non-protein nitrogen components like nucleic acids. A study by [107] measured TVB-N 28.95 mg/100 mL and explained that active packaging can preserve the quality of chicken meat because, in accordance with TVBN standards, chicken breast should not contain more than 60 mg/100 g of TVBN. Many factors can influence the migration of bioactive compounds from the film matrix to the food surface, including the amount of water in the food and the interaction between PLA and plant extracts or essential oils. Foods rich in water content can cause bioactive substances to migrate more quickly from the film matrix to the food surface. Additional factors such as film thickness and hydrophilicity may impact the rate at which bioactive substances migrate from the film matrix to the food surface [108,109,110,111].

## 6. The Future Trend of Smart Packaging Systems

Rice, poultry and poultry products, dairy, beverages, fruit, frozen foods, candy, and snacks are the most common food purchased. But fresh meat, vegetables, and fruits are most popular among workers nowadays as a result of the need for new and natural products as well as changes in consumer lifestyles, particularly during a global epidemic. However, the food industry faces the challenge of preserving the freshness of those products over long periods of storage. Many countries around the world have adopted active packaging technologies to some extent. The adoption of active packaging is not limited to a specific country but rather depends on the industry, market demand, and technological advancements. Countries with advanced food and pharmaceutical industries, such as the United States, Japan, Germany, and South Korea, have been early adopters of active packaging solutions. These countries often prioritize research and development in packaging technologies to improve product safety, shelf life, and consumer experience. However, active packaging concepts have been embraced to various degrees in other countries as well. According to the compound annual growth rate (CAGR), the smart packaging market is projected to reach USD 18.67 billion by 2028, growing at a CAGR of 6.55% during the forecast period (2023–2028). In 2023, the market was worth more than USD 13.59 billion. The need for smart and innovative packaging systems is not only limited to foodstuff. The cosmetic and skincare industry has also started to apply smart packaging for commercialized products. They focus on promoting product packaging that can be recycled at room temperature as a marketing strategy to attract consumer interest.

The production of smart packaging faces a common challenge, some of which are related to the kinetics of agent release, the compatibility of polymers and additives, and interactions between substances; therefore, it can compete with the characteristics of conventional plastics. Moreover, the implementation of smart packaging will also face the following techno-economic challenges:

1. Cost
2. Developing and incorporating smart packaging can be expensive, potentially increasing the overall cost of production and affecting product pricing.
3. Compatibility: Ensuring compatibility between different components of smart packaging, such as sensors and communication systems, can be challenging.
4. Data Security: Smart packaging often collects and transmits data, raising concerns about data security, privacy, and potential breaches.
5. Regulations: Compliance with regulatory standards and certifications can be intricate, especially in industries like pharmaceuticals and food, where safety is crucial.
6. Consumer Acceptance: Introducing new technology to consumers may require education and demonstration to ensure their understanding and willingness to use smart packaging.
7. Sustainability: Balancing the integration of electronics with sustainable and recyclable packaging materials can be challenging.
8. Technical Reliability: Ensuring the reliability and accuracy of sensors and communication systems over the entire product lifecycle can be complex.

Addressing these challenges requires collaboration among researchers, experts, technologists, and manufacturers in smart packaging to develop cost-effective, reliable, and user-friendly smart packaging solutions.

## 7. Conclusions 

The integration of smart technologies into PLA-based packaging enhances its functionality and value. Smart packaging can incorporate features like sensors and indicators to monitor various aspects of the packaged product, such as temperature, freshness, and authenticity. These capabilities offer several benefits, including improved supply chain visibility, enhanced product safety, and reduced food waste. The influence of natural extracts and essential oils on PLA-based smart packaging can be examined through their impact on the material’s physical, mechanical, and structural properties along with interfacial adhesion as well as their role in colony reduction. The efficacy of plant extracts, encompassing active agent amount, type, and PLA blend formulation will influence physical, mechanical, and colony reduction; their properties hinge on their chemical bond and interfacial adhesion with the PLA matrix. This interfacial adhesion can be optimized through techniques such as surface modification, compatibilizers, and encapsulation methods, ensuring a stable and controlled release of the active agents over time. Achieving effective chemical bonds and interfacial adhesion between the active agents and the PLA matrix is pivotal for unlocking the full potential of these enhancements and ensuring the sustained performance of the smart packaging system. Achieving a strong bond between the active agents and the PLA substrate is crucial for the consistent and prolonged release of bioactive compounds, thus prolonging perishable food shelf life. As the field of advanced materials continues to evolve, this integration holds significant promise for revolutionizing the packaging industry by providing sustainable, intelligent, and bioactive solutions. In confrontation to the complex challenges of the twenty-first century, the role of PLA in shaping the future of packaging emerges as a sign of hope, offering innovative solutions that not only improve food safety and preservation but also significantly contribute to a greener and more sustainable environment. This review serves as a timely reminder of the exciting possibilities that await in the field of smart packaging, strengthening PLA’s position as a key player in the evolution of packaging materials and strategies.

## Figures and Tables

**Table 1:** Common natural extract plants usually used to develop smart packaging.

Plant	Active Components
Jamun	β-humulen, α-guaiene, Caryophyllene, α-humulene, β-elemene
Propolis	dihydrochrysin, pinostrobin, caryophyllene and chrysin
Green tea	epigallo-catechin gallate
Clove	eugenol, eugenyl acetate and caryophyllene
Turmeric	α-turmerone, β-turmerone and ar-turmerone
Cinnamon	cinnamaldehyde, camphor, cinnamyl-acetate, caryophyllene, trans α-bergamotene, caryophillene oxide, linalool, geraniol, bornyl acetate, eugenol, γ-elemene, α-copaene, guaiol, and α-cubebene
Lemon	limonene, p-mentha-3,8-diene, β-pinene, γ-terpinene, myrcene, sabinene, myrcene, and geranial
Cymbopogon	myrcene, limonene, citral, geraniol, citronellol, geranyl acetate, neral, and nerol
Thymol	p-cymene, γ-terpinene and thymol
Eucalyptus	1,8-cineol and α-pinene
Oregano	rosmarinic acid, linalool, thymol, carvacrol, tannins, flavonoids, triterpenes, phenol carvacrol, and thymol.
Syzygium aromaticum	eugenyl acetate, eugenol, and β-caryophyllene

**Table 2:** Tensile Properties of PLA based Smart Packaging.

PLA/Active Agent Composition (%)	Active Agent	Tensile Strength (MPa)		Elongation at Breaks (%)		Reference
		^a^	^b^	^a^	^b^	
98/2	green tea extract	12.52	10.29	260.11	121.95	[52]
95/5	carvacrol	26.8	16.4	267.3	194.9	[53]
97/3	clove essential oil	43.30	11.8	2.60	30.7	[54]
98/2	mango peel extract	57.77	46.48	6.77	14.31	[55]
99/1	thyme essential oil	2.90	3.90	11.33	23.19	[56]
95/5	mediterranean propolis extract	36.80	26.2	2.9	3.0	[57]
91/9	thyme essential oil	64.16	49.81	3.08	175.99	[58]
95/5	thymol	29.6	4.02	176.4	76.61	[59]
98/2	rice straw extract	34	34	6	3.4	[60]
99.5/0.5	pomegranate peel extract	88.7	67.92	47.3	69.04	[61]

^a^ control sample, ^b^ sample with active agent.

**Table 3:** Effect of different film composition on WVTR.

Film Composition	WVTR (g/m^2^/s × 10^−4^)		Effect on Film Properties	Reference
	a	b		
PLA-Cinnamon essential oil	0.345	0.793	cinnamon essential oil is hydrophobic and creates pores that absorb more moisture	[66]
PLA-Betel leaf ethanolic extract	0.410	0.30	betel leaf ethanolic extract boosts molecular cross-linking, which reduces hydrophilic functional groups and slows water migration	[67]
PLA/PBAT-Peppermint essential oil	0.916	1.036	peppermint essential oil reduces the structural cohesiveness of the mixed film, allowing moisture to pass through the film more easily	[68]
PLA-Rosemary essential oil	1.70	1.58	the strong hydrophilicity of the biopolymer is attributed to a slight decrease in the water vapor barrier properties	[69]
PLA-Carvacrol essential oil	0.045	0.043	carvacrol essential oil (CEO is primarily made up of nonpolar hydrocarbon atoms (C-H) in the liquid phase, which makes nonpolar permeant molecules able to move around	[70]
PLA-PEG	6.28	6.44	PEG is hydrophilic; as the contact angle value rises, the hydrophilic properties also rise proportionally, and the contact angle value decreases water permeability	[71]
PLA/PBSA	0.175	0.129	PBSA crystalizes to create diffusion pathways for oxygen gas molecules, thus increasing the barrier of films	[72]
PLA/PBAT-Trans-cinnamaldehyde	0.154	0.169	PLA and trans-cinnamaldehyde have an intense interaction, leading to a plasticizing effect and an increase in free volume, which increases WVTR	[73]
PLA-Pea Starch	0.22	0.27	higher pea starch loading makes it easier for water molecules to saturate the bilayer films’ surface through hydroxylated PS/PLA chains and then enter the films through the spaces between starch molecular chains	[74]
PLA-Chitosan	3.75	0.085	chitosan is hydrophilic and has poor water vapor barrier properties; a higher amount of it causes the WVTR to increase	[75]
PLA/PHB-Cinnamaldehyde	0.26	0.69	cinnamaldehyde aldehyde group’s hydrophilicity resulting in higher WVTR	[76]
PLA-Oregano Essential Oil	0.112	0.135	the WVTR of PLA composite films explains how the presence of oregano essential oil makes the average film pore size larger	[77]

**Table 4:** Inhibitory effects of active agent additions.

Polymers	Inhibitory Effect	Reference
PLA-pink pepper essential oil	Pink pepper essential oil contains myrcene, which has antimicrobial action against *S. aureus* and *L. monocytogenes*, resulting in an inhibitory effect of 30 and 62%for *L. monocytogenes* and *S. aureus*, respectively, on day 21 of storage.	[86]
PLA-d-Limonene essential oil	Regardless of irradiation source or d-limonene loading, PLA/limonene films demonstrated 99.99% efficiency against *Escherichia coli*.	[87]
PLA-Polyphenols quercetin	The antibacterial level of reducing bacterial colonies against *Escherichia coli* films based on PLA increased to 87.8% with the addition of the polyphenol quercetin.	[88]
PLA-Ginger Essential Oil	The bacterial growth of the PLA/Ginger Essential Oil composite film was gradually stopped because of the presence of α-zingiberene and β-sesquiphellandrene.	[89]
PLA-Carvacrol essential oil	Carvacrol-containing films inhibited the growth of *Rhizopus* sp. and *Penicillium* sp.	[90]
PLA-Argan essential oil	The addition of argan essential oil was able to reduce the bacterial colonies of *E. coli* (86.5%), *L. monocytogenes* (72.2%) and *S. Typhimurium* (81.9%).	[91]
PLA-Persicaria hydropiper extract	The antibacterial activity of the ethanol extract of Persicaria hydropiper was able to reduce the growth of *S. aureus* (12.5%) but was unable to reduce the growth of *E. coli* and *S. Typhimurium*.	[92]
PLA-Oregano essential oil	The growth inhibition of *S. Typhimurium*, *E. coli*, and *L. monocytogenes* was up to 99%, after the addition of oregano oil stopped the growth of pathogenic bacteria in vitro.	[93]
PLA-Thyme essential oil	*E. coli* growth was slightly inhibited by thyme oil film (2.76%).	[94]
PLA-Allium ursinum extract	The antimicrobial activity of allium ursinum extract reduced colony growth of *S. aureus* (53%) and *E. coli* (100%)	[95]

**Table 5:** The effects of active agent additions on perishable food shelf-life quality.

Polymers	Methodology	Activity	References
PLA-Lemon extract	Lipid Oxidation Assays of almond including the following: Thiobarbituric acid-reactive substances (TBARS), Fat extraction, Peroxide value, *p*-Anisidine value.	The phenolic compounds in lemon extract improved the effectiveness of the film in preventing lipid oxidation in almonds kept at 40 °C for 30 days (83.33%).	[99]
PLA-Olive Pomace Extract	Physicochemical parameters (hardness, weight loss, and color) were evaluated after 12 days of storage at 4 °C.	Olive pomace extract maintained or increased the fruit’s total phenolic index and antioxidant potency while having no effect on firmness.	[100]
PLA-Lippia citriodora essential oil	The Quality Index Method (QIM) was used to perform sensory analysis on the rainbow trout fillet skin appearance (shiny to dull), the color of the fillets (pink to dark pink), the odor (freshness, seaweed, sour and rancid), and the texture (firm, elastic, soft, and very soft).	A score of “excellent” was given, and Lippia citriodora essential oil had no adverse effects on the sensory qualities of fish fillets.	[101]
PLA-Perilla essential oil	Kjeldahl distillation was used to determine the TVB-N content of chicken breast fillets.	This film increased the shelf life of chilled chicken by up to 12 days, as measured by a total volatile base nitrogen (TVB-N) 28.95 mg/100 mL assessment.	[102]
PLA-Marjoram essential oil	The total volatile base nitrogen (TVB-N) content of meat samples was determined using the AOAC (Association of Official Analytical Chemists) method.	A reduction of 1 log CFU/g of bacteria in beef was observed between the group that used marjoram essential oil.	[103]
PLA-Oregano essential oil	TVC was calculated to track when minced fish began to deteriorate microbiologically (TVC > 7 log cfu/g). Thiobarbituric acid (TBA) based on Malondialdehyde (MDA) value and Sensory evaluation (acceptability test) was performed using a hedonic scale point from 9 (most liked) to 1 (least liked) for minced fish.	After the sixth day of storage, the MDA value was concluded to be useless, because the TVC reached or exceeded the limit value of 7 log cfu/g.	[104]
PLA-Green tea extract	Smoked salmon was tested based on fat extraction to examine its peroxide value, *p*-Anisidine value and TBARS.	Aldehydes were present, as indicated by the p-anisidine value, and TBARS demonstrated a 33% reduction in aldehyde.	[105]

## Data Availability

Not applicable.
